# Supplementary material for: Vascularized tumor on a microfluidic chip to study mechanisms promoting tumor neovascularization and vascular targeted therapies
Source: Theranostics. 2025 Jan 1;15(3):766–83. doi: 10.7150/thno.95334 (PMC11700857; doi:10.7150/thno.95334)
Supplement: Supplementary file 1 — Supplementary figures and videos. [file thnov15p0766s1.pdf]

Supplementary Material: Vascularized tumor on a microfluidic chip to study mechanisms promoting tumor neovascularization and vascular targeted therapies

Short title (50 characters): Microfluidic chip study of tumor vascularization

Magdalena Skubal[1], Benedict Mc Larney[1], Ngan Bao Phung[1,3], Juan Carlos Desmaras[1], Abdul Vehab Dozic[1], Alessia Volpe[1,2], Anuja Ogirala[1], Camila Longo Machado[1,2], Jakob Djibankov[1], Vladimir Ponomarev[1,2,3,4], Jan Grimm[1,2,3,4,*]

[1] Molecular Pharmacology Program, Memorial Sloan Kettering Cancer Center, New York, NY, USA
[2] Department of Radiology, Memorial Sloan Kettering Cancer Center, New York, NY, USA
[3] Department of Pharmacology, Weill Cornell Medical College, New York, NY, USA
[4] Department of Radiology, Weill Cornell Medical College, New York, NY, USA
[*]Correspondence: Jan Grimm grimmj@mskcc.org

keywords: microfluidic chip, neovascularization, targeted therapies, confocal imaging, optoacoustic imaging

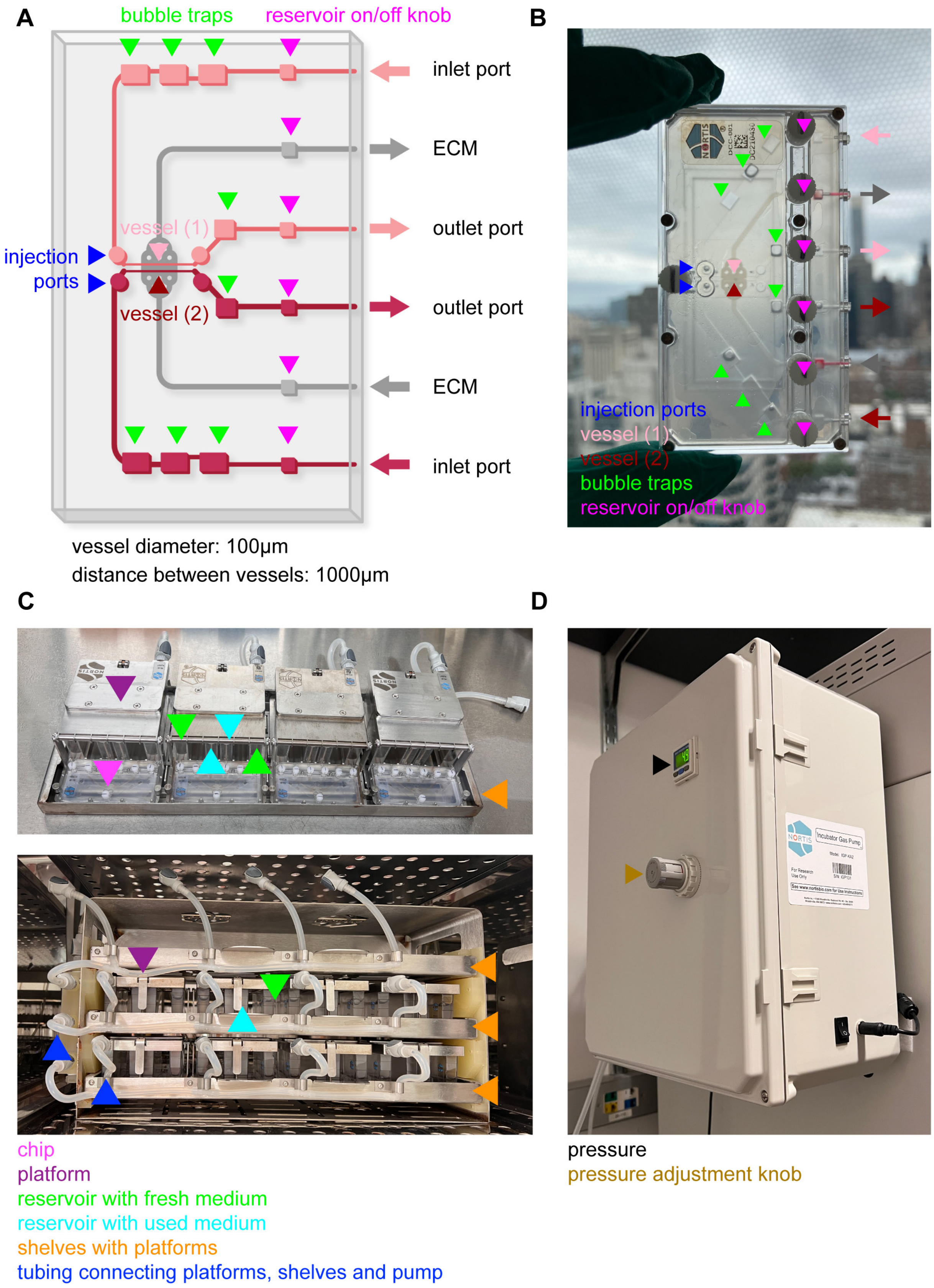
A
bubble traps
reservoir on/off knob
inlet port
ECM
outlet port
injection
ports
vessel (1)
vessel (2)
outlet port
ECM
inlet port
vessel diameter: 100µm
distance between vessels: 1000µm
B
injection ports
vessel (1)
vessel (2)
bubble traps
reservoir on/off knob
C
D
chip
platform
reservoir with fresh medium
reservoir with used medium
shelves with platforms
tubing connecting platforms, shelves and pump
pressure
pressure adjustment knob

Figure S1. Double channel microfluidic chip. *(A) Schematic visualization and* ***(B)*** *image of double channel MFC built of ECM (extracellular matrix), inlet (fresh medium) and outlet (used medium) ports, vessels, injection ports, bubble traps, reservoir on and off knobs.* ***(C)*** *Single shelf comprised of 4 MFC platforms (top) and image of the MFC system comprised of 3 shelves with 4 chips per shelf within the incubator (bottom). Platform locations, connection tubing, shelving, fresh and used media reservoirs are highlighted.* ***(D)*** *Pumping unit mounted on a dedicated incubator with pressure indicator and pressure adjustment knob.*

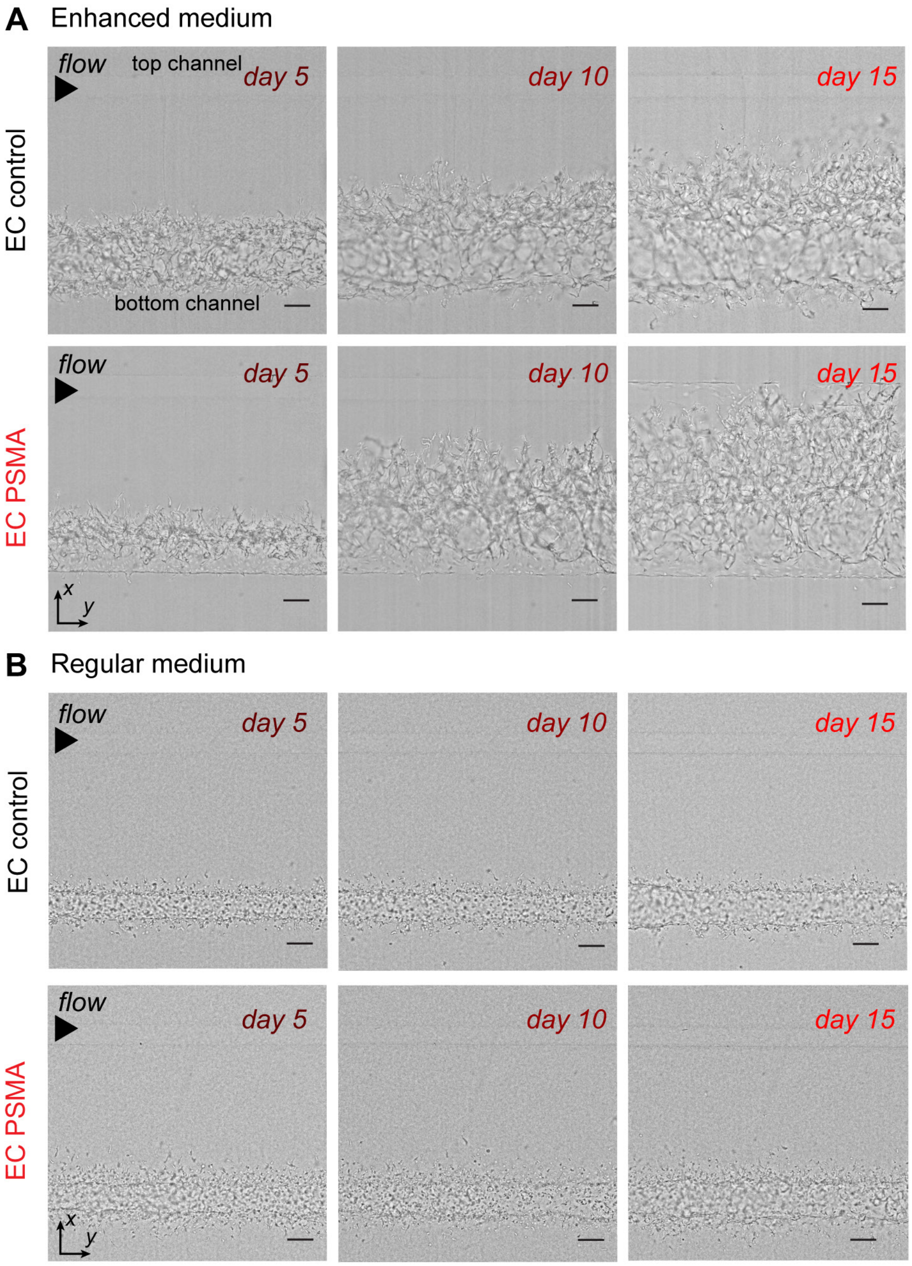

Figure S2. *(A) Confocal microscopy images showing sprouting of EC control and EC PSMA at days 5, 10 and 15. Regular medium was perfused through the lower channel seeded with EC, while*

enhanced medium supplemented with PMA and additional growth factors (VEGF and FGF) was perfused through the upper channel. Scale bars 100 µm. (**B**) Confocal microscopy images showing sprouting of EC control and EC PSMA cells on days 5, 10 and 15, where regular medium was perfused through both channels. Scale bars 100 µm.*

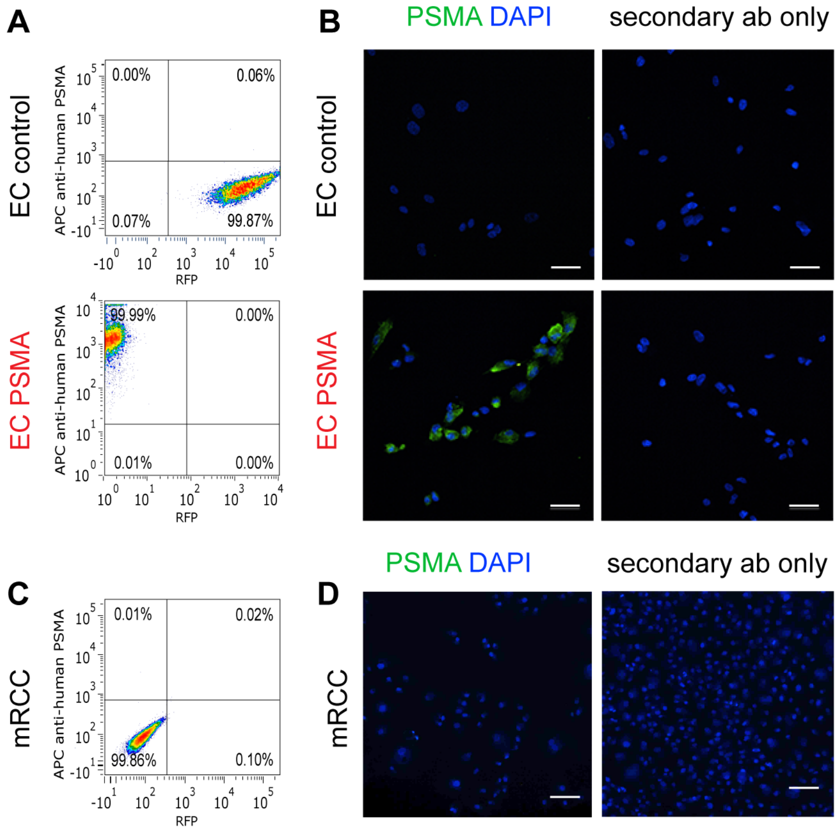

***Figure S3. PSMA expression in endothelial and renal carcinoma cells.** (A, **C**) Dot-plots from flow cytometric analysis and (**B**, **D**) immunostaining analysis confirming PSMA expression in control EC (EC control), EC engineered by retroviral gene transfer to stably overexpress functional PSMA protein (EC PSMA) and renal carcinoma cells (mRCC). EC control and mRCC are negative for PSMA. Scale bars 100 µm.*

A Automated data processing:

1. Acquiring images at days 5, 10, 15
2. Convertion of images from .nd2 to .tiff
3. Determintion of ROI and ROI center
4. Devision of ROI to 20 eqal parts
5. Quantfication of the area occupied by EC

B

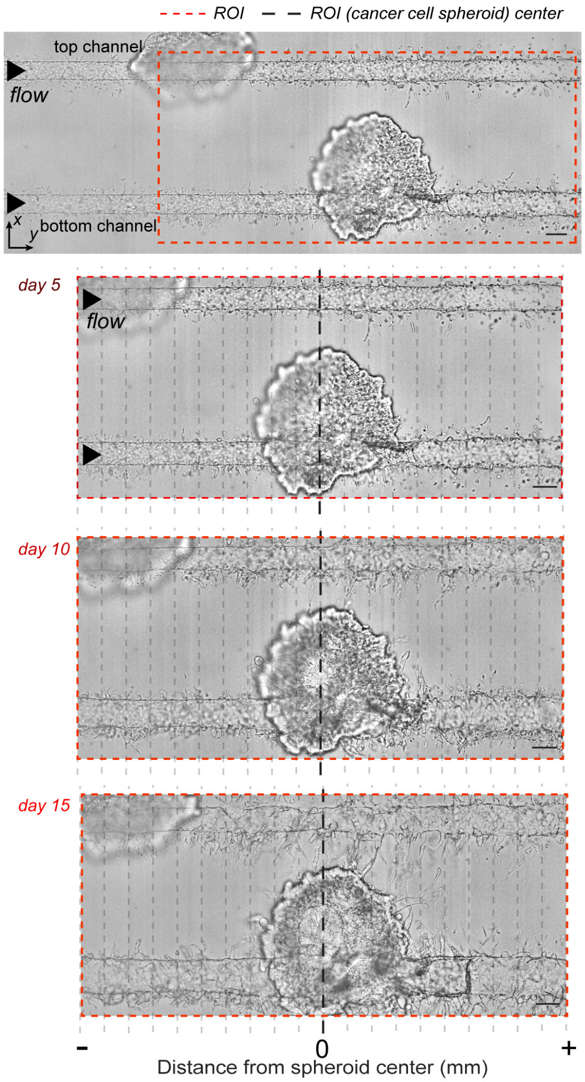

C

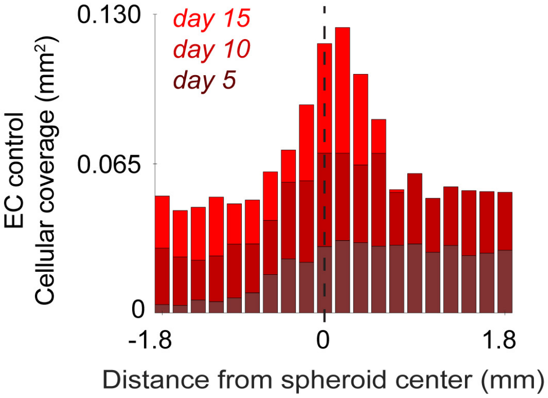

Figure S4. Quantification of cellular coverage induced by mRCC spheroids. *(**A**) Steps applied to quantify EC coverage: coverage of EC was quantified in the region of interest (ROI) labeled by red dotted lines, the ROI was divided into 20 respective equidistant sections in all cases labeled by gray dotted lines, the ROI center is labeled by black dotted lines and the area occupied by sprouting EC was quantified in relation to the ROI center. (**B**) Representative images of EC and mRCC spheroid co-culture maintained on chip at days 5, 10 and 15. Scale bars 100 µm. (**C**) Quantification of the area covered by EC sprouting induced by mRCC spheroid.*

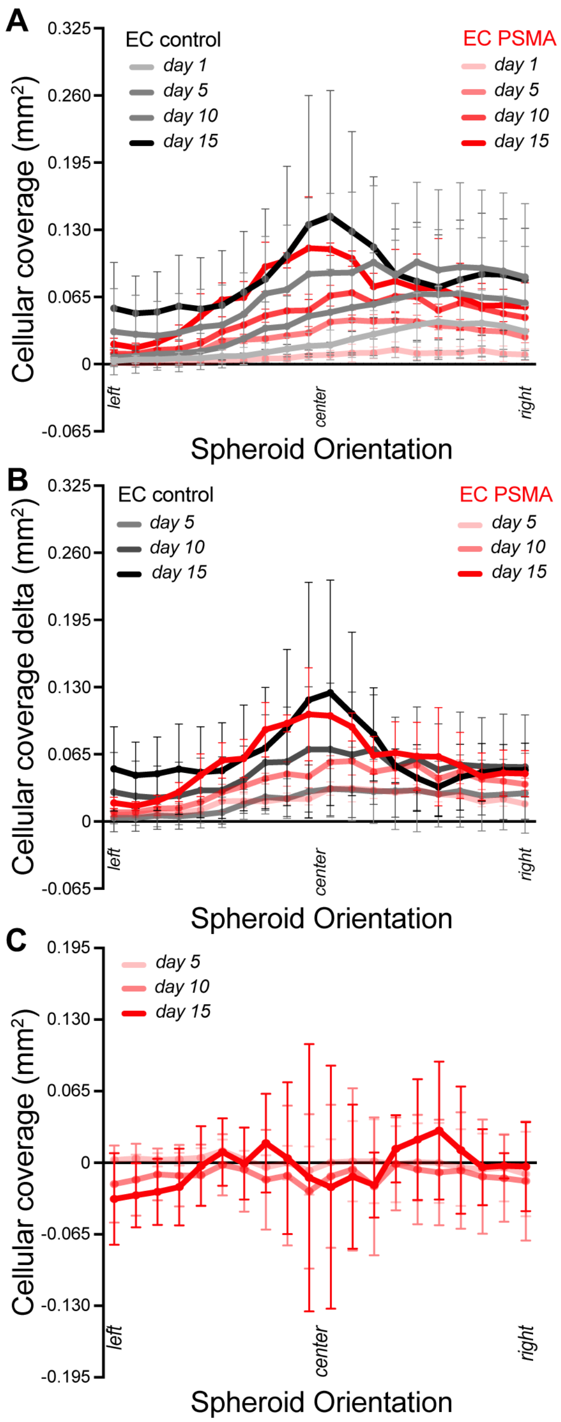

Figure S5. Cellular coverage of EC control and EC PSMA. *(**A**) Plots showing the area covered by EC control and EC PSMA sprouting towards mRCC spheroid at days 1, 5, 10 and 15. (**B**) Plots showing the area occupied by EC control and EC PSMA sprouting towards mRCC at days 5, 10 and 15 after subtraction of day 1 seeding coverage from each respective chip from all other respective timepoints. (**C**) Cellular coverage of EC control coverage values subtracted from EC PSMA values to assess the difference between the groups. No statistically significant regions or timepoints were identified highlighting the EC control similarity to EC PSMA also in line with the*

*induced PSMA expression (see main Figure 2D-E). For panels (**B**) and (**C**) subtraction of Day 1 EC seeding enables counteraction of flow effects.*

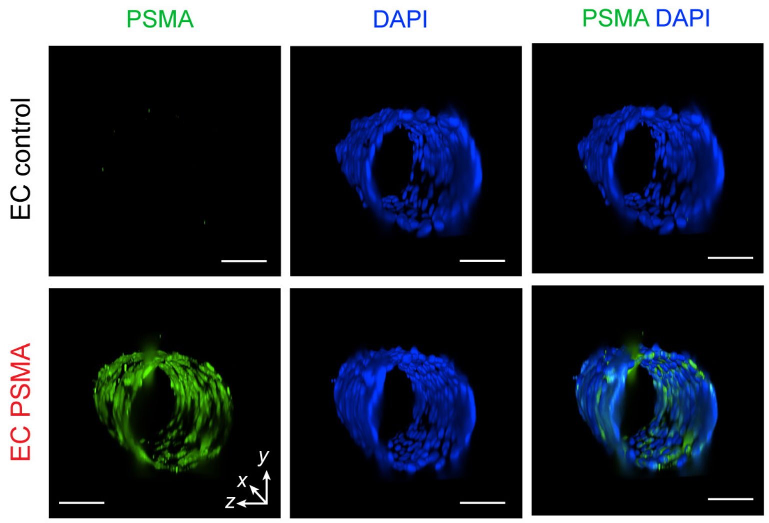

Figure S6. PSMA expression in EC on microfluidic chip. *Immunostaining confirming PSMA expression in EC PSMA (n = 1) engineered to stably overexpress functional PSMA protein, and no PSMA expression in EC control (n = 1) at day 1. Scale bars 50 µm.*

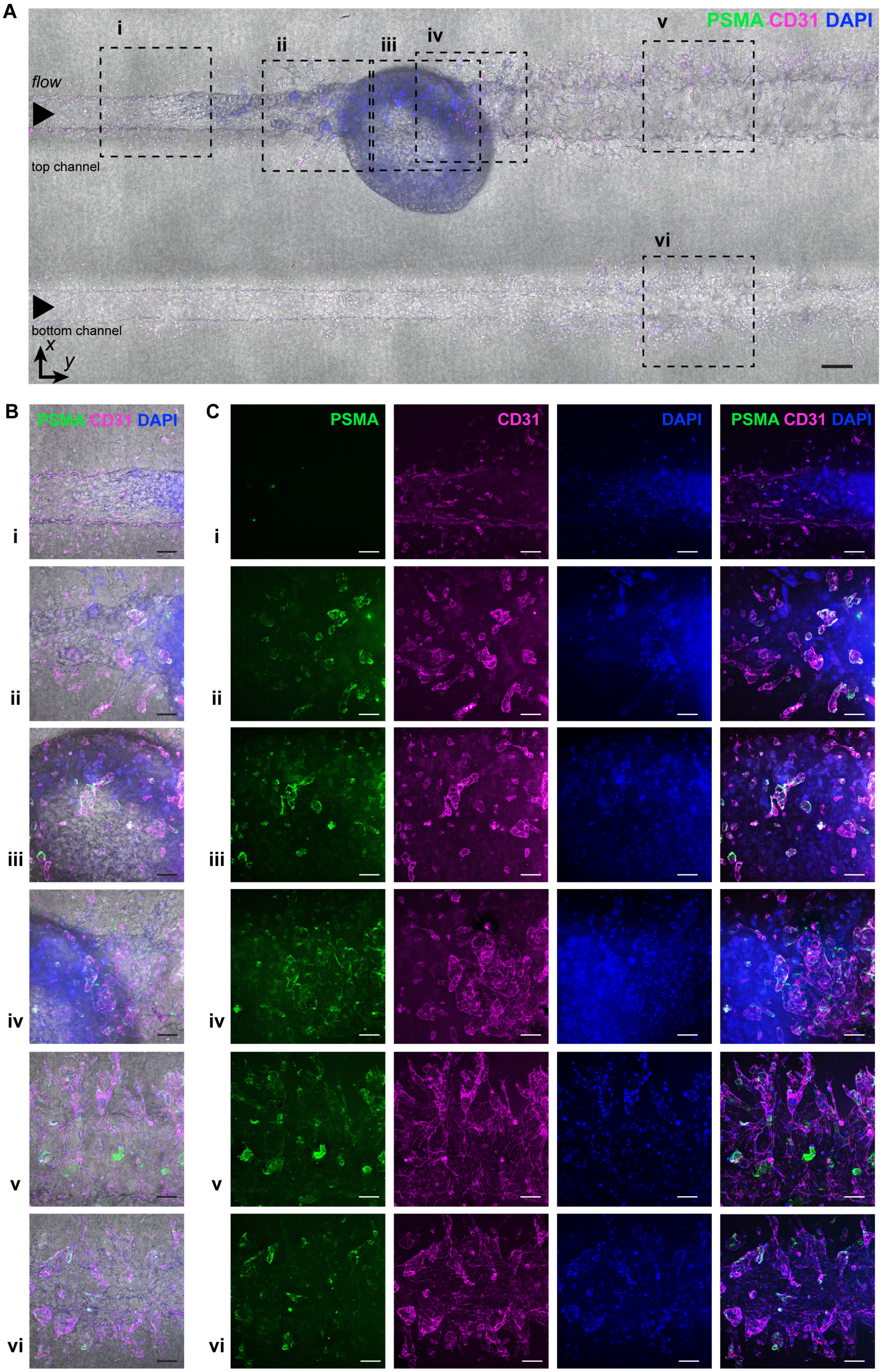
A
PSMA CD31 DAPI
i
ii
iii
iv
v
vi
flow
top channel
bottom channel
x
y
B
PSMA CD31 DAPI
C
PSMA
CD31
DAPI
PSMA CD31 DAPI

Figure S7. Induced PSMA expression on tumor associated neovasculature. *(A-C) Immunostaining showing PSMA (green) expression induced on control EC (CD31, magenta) co-cultured with mRCC spheroids for 15 days on the MFC. Endothelial and cancer cell nuclei stained with DAPI (blue). Scale bars 100 µm.*

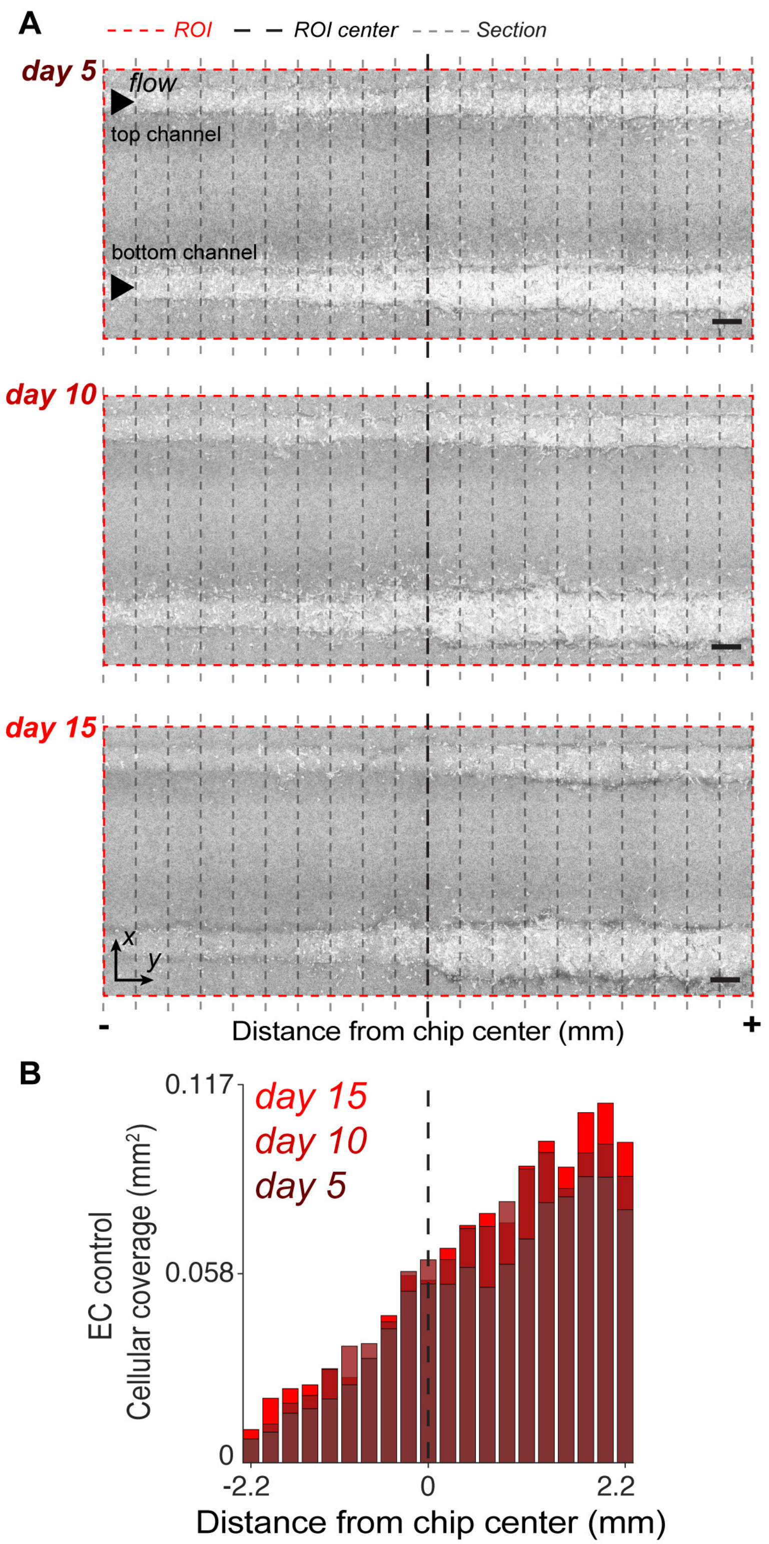

Figure S8. Quantification of cellular coverage in response to flow effect. *(**A**) Representative images of EC control maintained on chip at days 5, 10 and 15 to visualize flow effects. Scale bars 100 µm. Note that the flow direction is from left to right and appears to influence EC seeding. (**B**)*

Quantification of the area covered by EC sprouting in response to flow effect (n = 3). Coverage of EC was quantified in the same manner as in Figure S3 A.

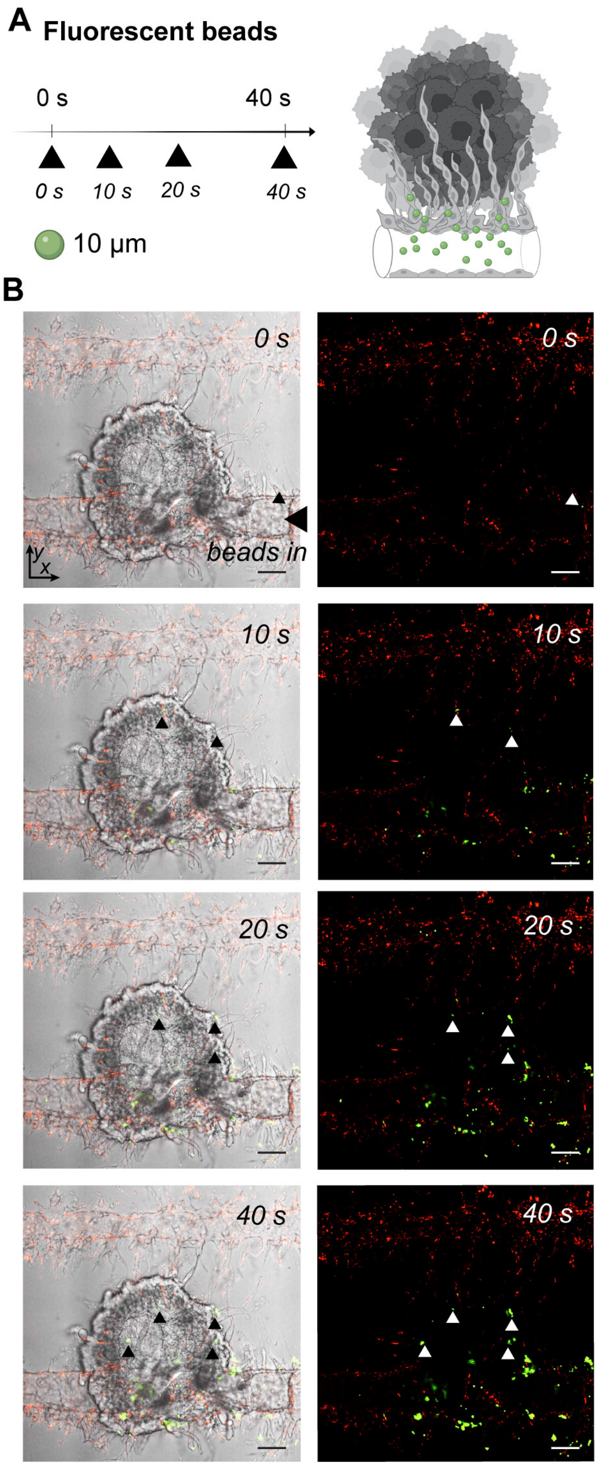

Figure S9. Mimicking blood flow on the microfluidic chip. *(**A**) Functionality of tumor associated blood vessels was tested by perfusion of ø10 µm green, fluorescent beads through tumor associated blood vessels. (**B**) Beads injected into tumor on chip (n = 1) with well-developed vasculature (day*

15) through the outlet port, entered chip channels and newly formed vessels surrounding the surface of the mRCC spheroid (right panel: beads with EC surrounding mRCC spheroid, left panel: beads with EC) at 0 s, 10 s, 20 s and 40 s. Scale bars 100 µm.

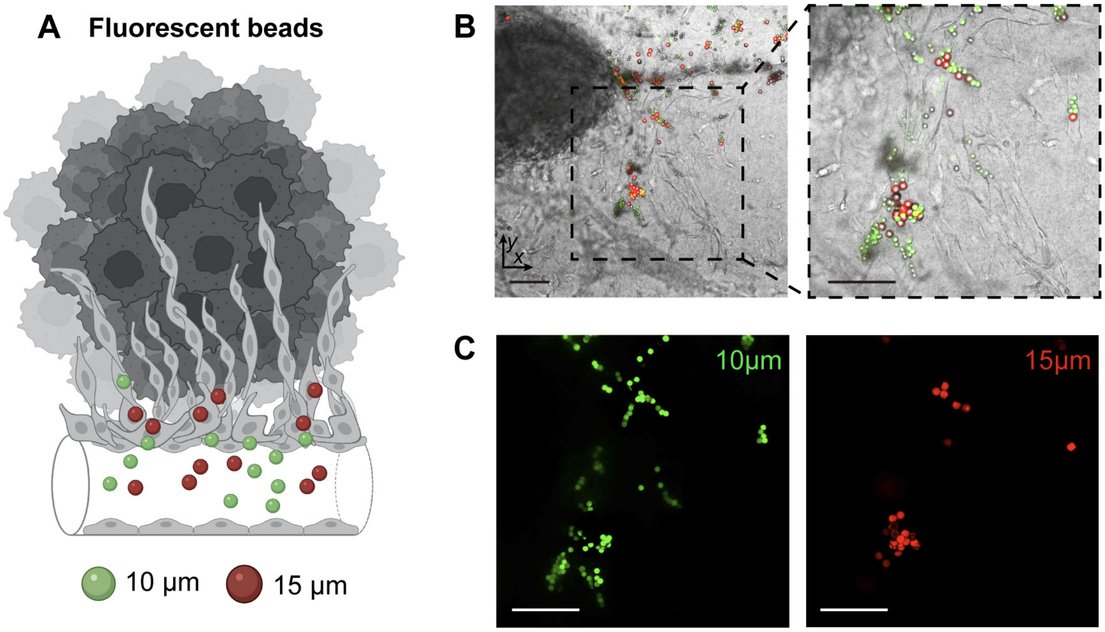

***Figure S10. Mimicking blood flow on the microfluidic chip.** (**A**) Functionality of tumor associated blood vessels was tested by perfusion of ø10 µm (green) and ø15 µm (red) fluorescent beads representing sizes in immune cells. Beads injected into tumor on chip (n = 1) with well-developed vasculature (day 15) through the outlet port, entered chip channels and newly formed vessels surrounding the surface of the mRCC spheroid. (**B**, **C**) Confocal microscopy images of 10 µm and 15 µm beads traveling through the chip. Scale bars 100 µm.*

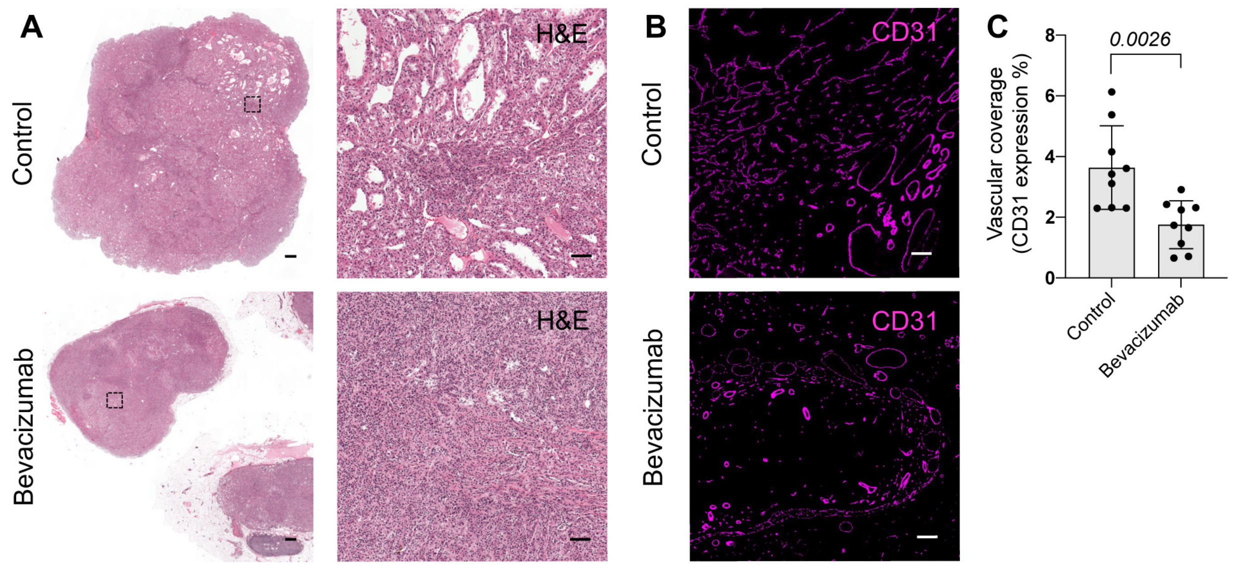

Figure S11. Vascular targeted therapy in vivo. *Immunostaining showing responses to vascular targeted therapy in mRCC tumor model evaluated by treatment with bevacizumab. (**A**) Representative hematoxylin and eosin staining of control and bevacizumab treated tumors. Scale bars 1000 µm and 100 µm, respectively. (**B**) Representative images of CD31 staining of control and bevacizumab treated tumors. Scale bars 100 µm. (**C**) Quantification of vascular coverage after bevacizumab treatment. Displayed p values are based upon a two-sided Student's t test.*

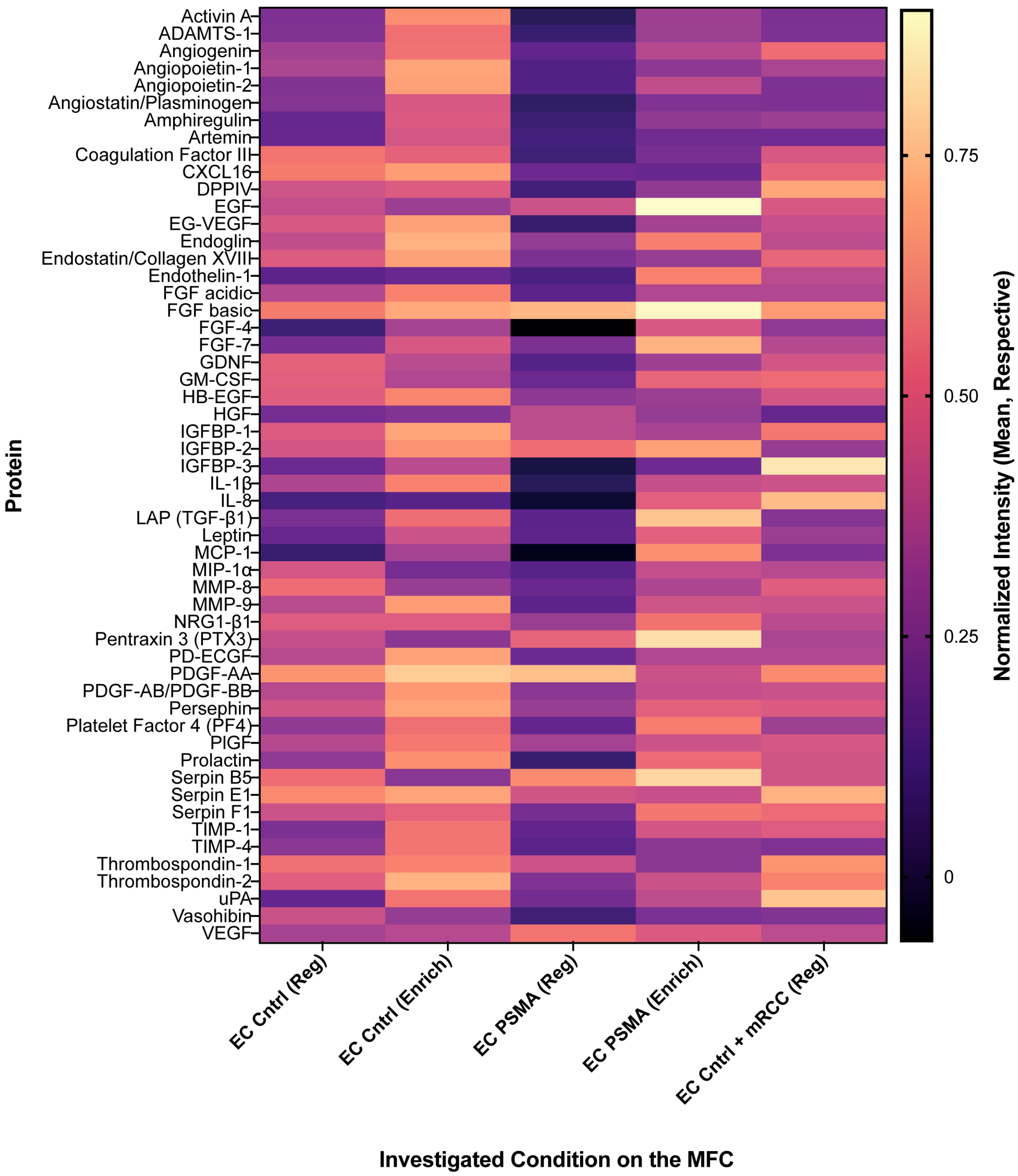

Figure S12. Microfluidic system and assessment of angiogenesis-related proteins. *Heatmap shows the mean values of 55 angiogenesis-related proteins expression in the following conditions: EC control perfused with regular medium (EC control (Reg), EC control perfused with enriched media (EC control (Enriched)), EC PSMA perfused with regular medium (EC PSMA (Reg)), EC PSMA perfused with enriched medium (EC PSMA (Enrich)) (n = 6 technical from n = 3 biological replicates for each condition), and EC control with mRCC spheroid perfused with regular medium (EC control + mRCC (Reg)). Protein expression levels were assessed after 15 days of growth, all protein expression has been background subtracted to account for array gel autofluorescence*

followed by normalization to reference points within the microarray to account for potential differences between imaging. Finally, to better elucidate differences between investigated conditions the values have been normalized via division of the highest expression condition.

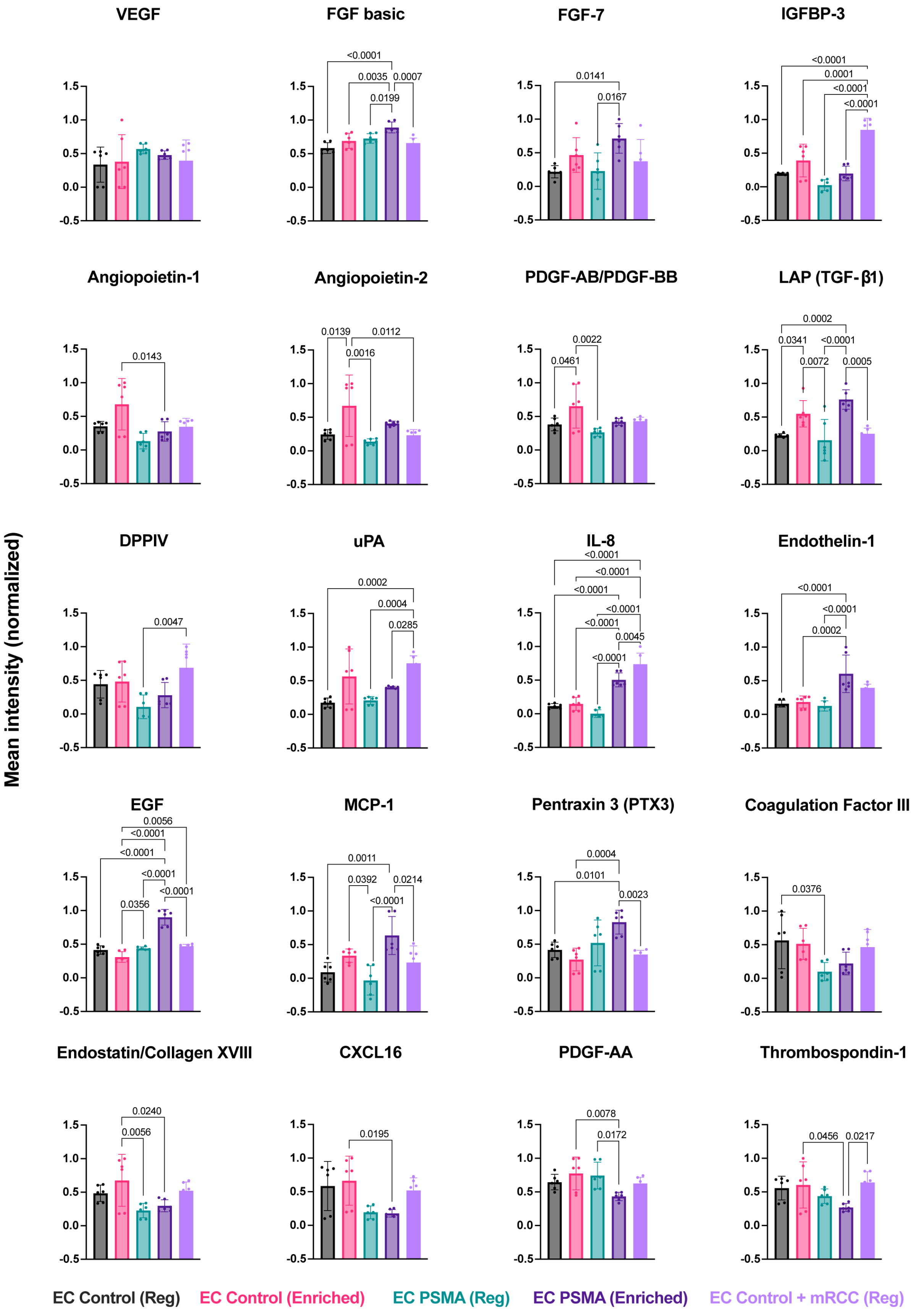

Mean intensity (normalized)
VEGF
FGF basic
FGF-7
IGFBP-3
Angiopoietin-1
Angiopoietin-2
PDGF-AB/PDGF-BB
LAP (TGF-β1)
DPPIV
uPA
IL-8
Endothelin-1
EGF
MCP-1
Pentraxin 3 (PTX3)
Coagulation Factor III
Endostatin/Collagen XVIII
CXCL16
PDGF-AA
Thrombospondin-1
EC Control (Reg)
EC Control (Enriched)
EC PSMA (Reg)
EC PSMA (Enriched)
EC Control + mRCC (Reg)

Figure S13. Expression level of assessed angiogenic factors from MFC system. *EC control + mRCC (Reg) group showed higher expression levels in DPPIV, uPA, and significantly higher in IGFBP-3, and IL-8 compared to other groups. EC Control (Enriched) group had higher expression level in 4 factors (angiopoietin-1,2, PDGF-AB/PDGF-BB, and LAP(TGF- β1), EC PSMA (Enriched) had significantly higher expression level in the following 8 factors: FGF-7, EGF, endothelin-1, FGF basic, LAP(TGF-β1), MCP-1, IL-8 and pentraxin 3 (PTX3) compared to most groups. Compared to EC control (Enriched), EC PSMA (Enriched) had lower expression level of the following proteins: CXCL16, endostatin/collagen XVII, PDGF-AA, and thrombospondin-1. Displayed significant p values are based upon ordinary one-way ANOVA with Tukey's multiple comparison test (n = 6 technical from n = 3 biological replicates for each condition)*

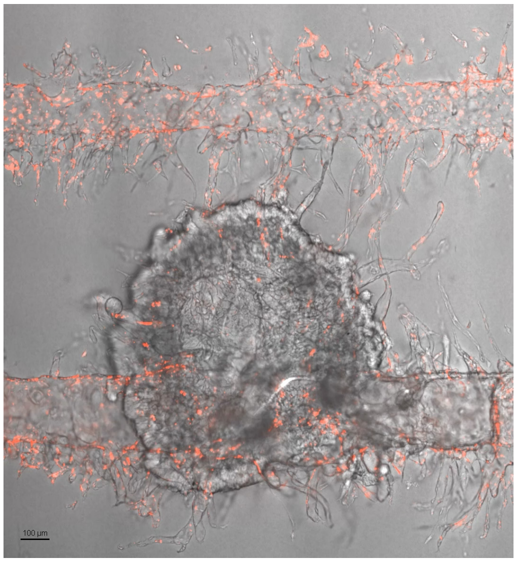

Video 1: Mimicking blood flow. *Fluorescent ø10 µm beads (green) perfused through vascularized tumor on a microfluidic chip at day 15 (EC: red, mRCC spheroid: grey).*

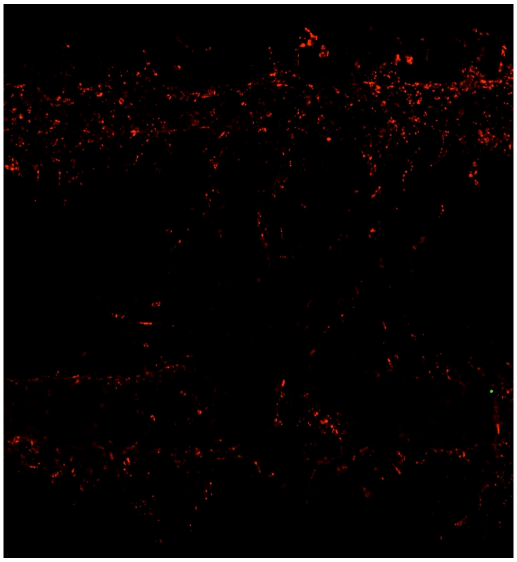

Video 2: Mimicking blood flow. *Fluorescent ø10 µm beads (green) perfused through vascularized tumor on a microfluidic chip at day 15 (EC: red).*

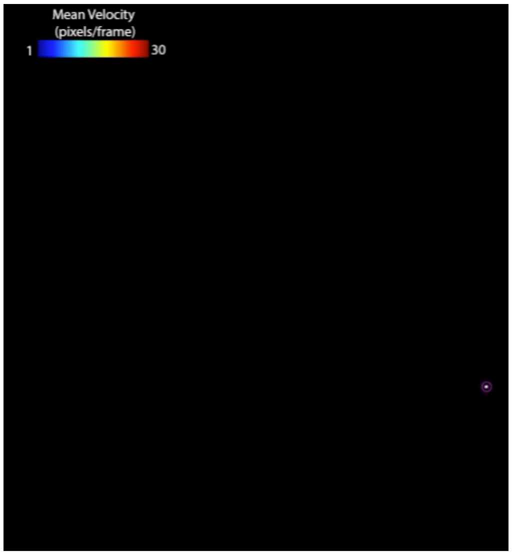

Video 3: Mimicking blood flow. *Tracking of fluorescent ø10 µm beads perfused through vascularized tumor on a microfluidic chip at day 15.*

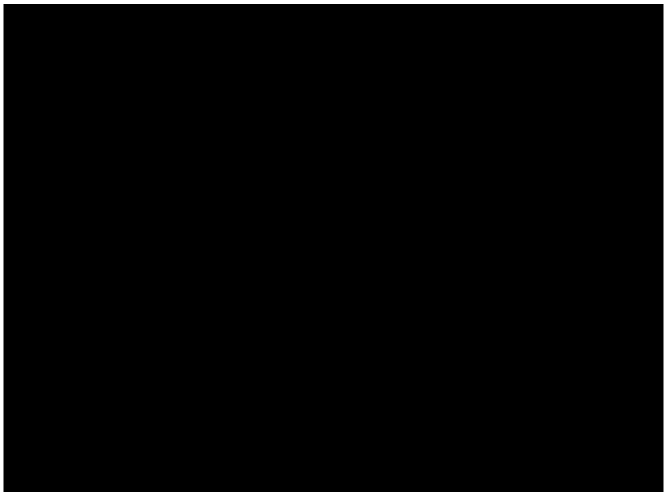

Video 4: Mimicking drug delivery. *Fluorescein (green) perfused (washed in) through vascularized tumor on a microfluidic chip at day 5 (EC: red, mRCC spheroid: grey).*

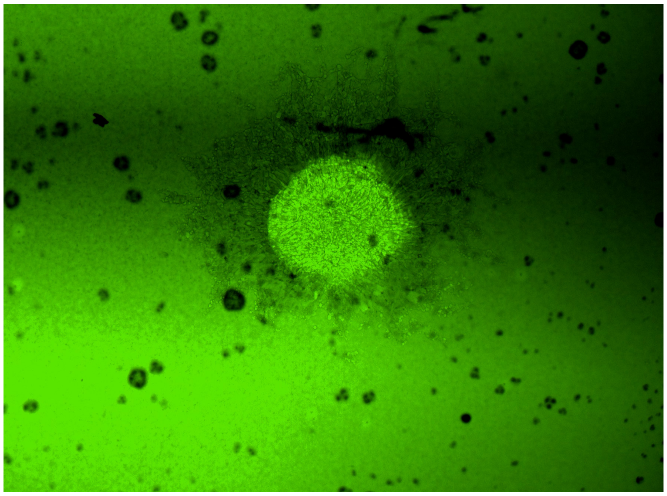

Video 5: Mimicking drug delivery. *Fluorescein (green) perfused (washed out) through vascularized tumor on a microfluidic chip at day 5 (EC: red, mRCC spheroid: grey).*
